# Surgical treatment of atlantoaxial subluxation by intraoperative skull traction and C1-C2 fixation

**DOI:** 10.1186/s12891-020-03273-7

**Published:** 2020-04-14

**Authors:** Jianwei Guo, Wencan Lu, Xiangli Ji, Xianfeng Ren, Xiaojie Tang, Zheng Zhao, Huiqiang Hu, Tao Song, Yukun Du, Jianyi Li, Cheng Shao, Tongshuai Xu, Yongming Xi

**Affiliations:** 1 Department of Orthopedics, The Affiliated Hospital of Qingdao University, 16 Jiangsu Road, Qingdao, 266003 Shandong Province People’s Republic of China; 2 Department of Spine Surgery, Shenzhen University General Hospital, Xueyuan AVE 1098, Nanshan District, Shenzhen, Guangdong People’s Republic of China; 3 Department of Intensive Care Unit, Qilu Hospital of Shandong University (Qingdao), 758 Hefei Road, Qingdao, 266035 Shandong Province People’s Republic of China; 4 Department of Orthopedics, Yantai Affiliated Hospital of Binzhou Medical University, 717 Jinbu Street, Muping District, Yantai, 264000 Shandong Province People’s Republic of China

**Keywords:** Atlantoaxial subluxation, Reduction, Skull traction, General anesthesia

## Abstract

**Background:**

Atlantoaxial subluxation (AAS) is a not rare abnormality between the atlas (C1) and axis (C2). For AAS patients with persistent neck pain and neurologic symptoms, surgical intervention is a good choice. Nevertheless, there were still few reports about the use of intraoperative skull traction and different fixation methods in treatment of AAS.

**Methods:**

From January 2012 to December 2018, a total of 86 cases were admitted to our hospital and diagnosed as AAS. All the patients received atlantoaxial reduction with the help of intraoperative skull traction and C1-C2 fixation. Clinical and radiological parameters were collected through chart review.

**Results:**

There were 86 cases included in this study. The mean operative time was 153.9 ± 73.9 min, and the mean amount of intraoperative blood loss was 219.1 ± 195.6 ml. 81 patients underwent posterior reduction, internal fixation and fusion. 5 patients underwent anterior release, followed by posterior internal fixation and fusion. 82 patients got satisfactory postoperative outcomes while complications occurred in 4 patients. Significant neurologic improvement was observed in these patients. Bone fusion was achieved on the midline sagittal reconstructed CT images at the latest follow-up in all these patients except 1 case. All the patients were followed up for 34.84 ± 15.86 months at average (range 12–60 months). The mean ADI value was 7.55 ± 1.67 mm at average preoperatively, and improved to 4.03 ± 1.21 mm postoperatively, and to 4.21 ± 0.99 mm at the latest follow-up. The mean A-A angle was 15.48 ± 9.82 degrees at average preoperatively, and improved to 21.61 ± 10.43 degrees postoperatively, and to 19.73 ± 8.13 degrees at the latest follow-up. The mean A-A height was 35.61 ± 7.66 mm at average preoperatively, and improved to 40.08 ± 8.5 mm postoperatively, and to 38.83 ± 6.97 mm at the latest follow-up. There were complications in 4 patients, including pedicle misplacement, pedicle screw fracture, infection and one death.

**Conclusion:**

Intraoperative skull traction can effectively facilitate the surgical procedures for ASS caused by different etiologies. Further research will be needed to investigate the safety and effectiveness of this method in the future.

## Background

Atlantoaxial subluxation (AAS) is a not rare abnormality between the atlas (C1) and axis (C2). Several diseases have been reported to be associated with the occurrence and development of AAS, including inflammatory, congenital, traumatic, and neoplastic processes [1, 2]. These processes can damage the zygopophysis joint or ligament between the atlas (C1) and axis (C2), and cause excessive movement and instability at this junction, resulting in atlantoaxial subluxation. It can cause neck pain and spinal cord compression, even irreversible neurological deficits, such as cervical myelopathy, paresis, respiratory dysfunction, and even consequent death [3]. Early diagnosis and appropriate treatment should be done for this kind of abnormity.

For AAS patients with persistent neck pain and neurologic symptoms, surgical intervention is a good choice. Multiple procedures have been used to stabilize the atlantoaxial joints and achieve spinal cord decompression. These procedures were performed by fixation between C1 and C2 at either a lateral mass [4], a pedicle [5], a lamina of C2 [6], or transarticular screws [7]. These procedures have been reported to achieve good surgical outcome and radiological improvement in earlier reports. Besides, intraoperative skull traction has been proved to be a useful method in the reduction of AAS. Nevertheless, there were still few reports about the use of intraoperative skull traction and different fixation methods in treatment of AAS. Therefore, we conduct this study to evaluate the surgical outcomes and radiological improvement of AAS by using intraoperative skull traction and different fixation methods.

## Methods

### Patients

This study was approved by our hospital’s ethics committee. From January 2012 to December 2018, a total of 86 cases were admitted to our hospital and diagnosed as AAS. Among them, 53 cases were males and 33 cases were females, with the average age of 52.8 ± 14.3 years (17–83 years). All the patients met the following criteria: 1) complaint of neck pain and varying degrees of neurological defects; 2) radiological findings confirm the presence of AAS and spinal cord compression; 3) all the patients received intraoperative skull traction and atlantoaxial reduction and fixation; 4) all the patients got at least 1- year regular follow-up. Patients without intact follow-up data or follow-up time < 1 year or with atlantoaxial tumor or infection were excluded.

All the patients received cervical posteroanterior and lateral radiography, dynamic lateral radiography, three-dimensional CT, computed tomography angiography (CTA) of cervical arteries, and magnetic resonance imaging (MRI) of the cervical spine. The sex, age, pathology, operative time, blood loss, follow-up time and complications were collected in Table 1 through chart review.

**Table 1:** Patient demographic and clinical data

Demographics	
Sex (Femal/Male)	33/53
Age (Years)	52.8 ± 14.3
Pathology	
Rheumatoid arthritis	9
Basilar invagination (BI)	3
Old odontoid fractures	5
Os odontoideum	11
Acute cervical trauma	27
No specific reasons	33
Operative time (min)	153.9 ± 73.9
Blood loss (mL)	219.1 ± 195.6
Follow-up (months)	34.84 ± 15.86
Complications	4

### Surgical procedure

**Fig. 1:**
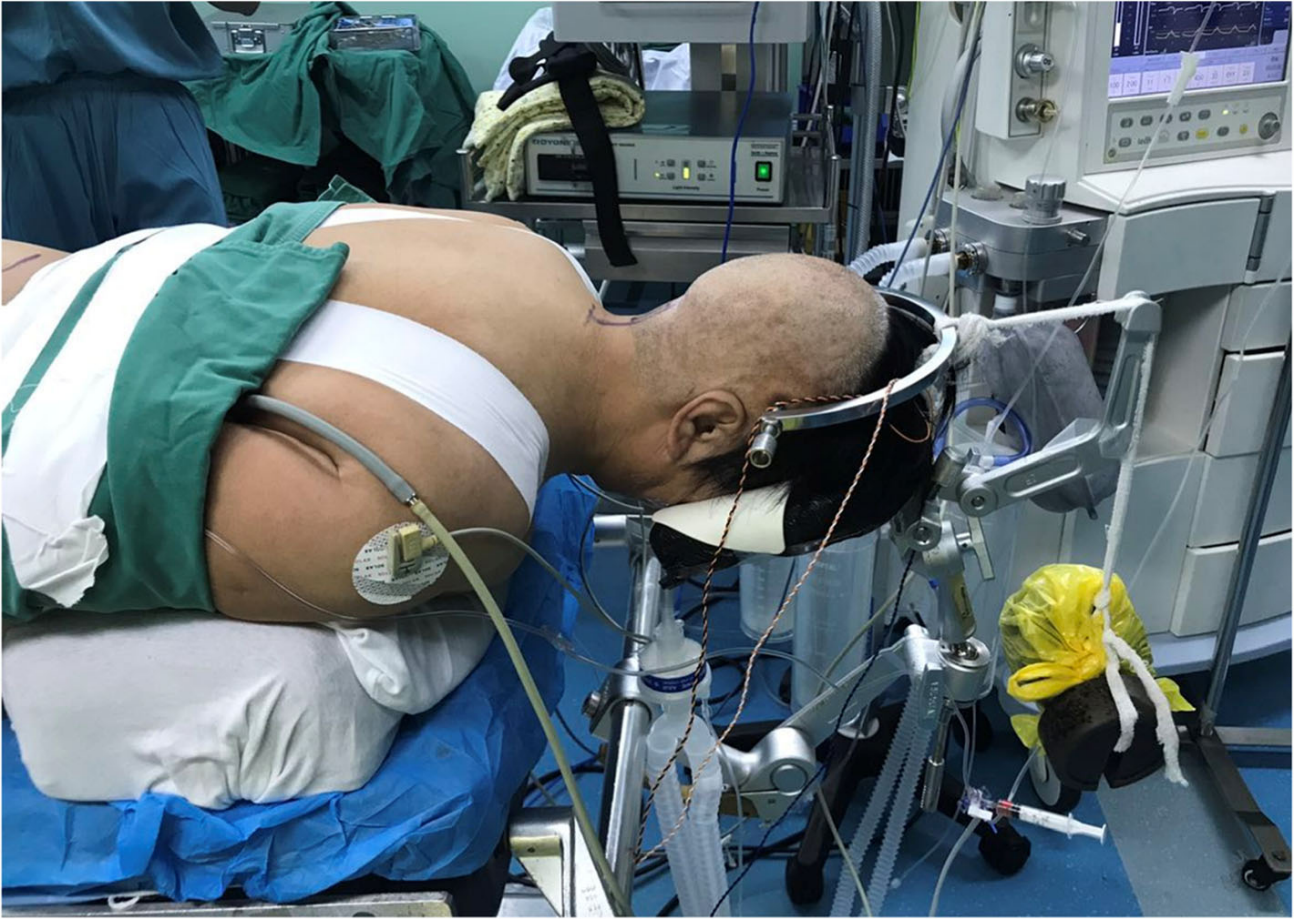
The patient was placed in the supine position. Gardner–Wells tongs traction was performed to observe the reduction of AAS. The initial traction was performed from 3 kg for 3 min, the traction weight would increase in accordance with the reduction of AAS, but no more than one-sixth to one-fifth of the patient’s weight

After general anesthesia, all the patients were placed in the supine position. Gardner–Wells tongs traction was performed to observe the reduction of AAS (Fig. 1). The initial traction was performed from 3 kg for 3 min, the traction weight would increase in accordance with the reduction of AAS, but no more than one-sixth to one-fifth of the patient’s weight. Somatosensory evoked potentials (SEPs) were used to monitor the neurologic signal throughout the traction procedure. Once the anatomic reduction was achieved, or further reduction could not be achieved with the maximum traction weight applied for 15 min, or abnormal SEPs were observed during traction, the traction procedure was terminated [8].

**Fig. 2:**
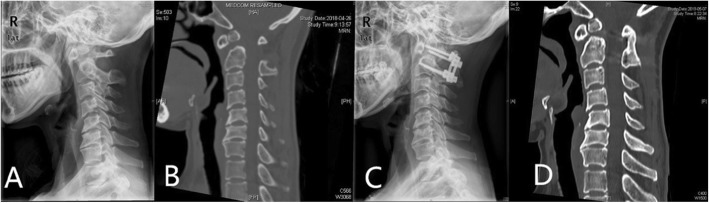
A 48-year old female suffered from progressive numbness and weakness in the left upper limb for 1 years. Sagittal X-ray (**a**) and sagittal reconstructed CT images of the cervical spine (**b**) showed os odontoideum and subluxation at the atlanto-axial joint. Posterior atlantoaxial reduction, fixation and bone graft fusion with intraoperative skull traction under general anesthesia were performed. Satisfactory reduction and fixation were achieved after surgery (**c**). Sagittal reconstructed CT images of the cervical spine (**d**) at 1-year follow-up confirmed good fusion at the atlanto-axial joint

81 cases achieved satisfactory reduction of AAS, and posterior-only C1-C2 internal fixation and fusion were performed (Fig. 2). These patients were turned into the prone position with the skull traction. The occipital squama, the posterior edge of the occipital foramen, the C1–C3 spinous process, and the lateral mass were exposed via a posterior approach. C1 lateral mass screws and C2 pedicle screws or laminae screws were implanted according to the C2 pedicle and HRVA. Two rods were bent to achieve suitable curve and were used to connect the screws at the same side. The C2 screw heads were tightened firstly, and then the C1 screw heads were tightened. If C1 screw heads could not be connected, the C1 spinous process would be lifted up or the C2 spinous process would be pressed downwards. And then all the screws were tightened. The cortical bone at C1–2 was removed to achieve the bone graft bed, and the iliac or allogenic cancellous bone was grafted finally.

In 5 cases, satisfactory reduction could not be achieved, anterior release surgery and posterior C1-C2 internal fixation and fusion were needed. The patients were placed in a supine position with continuous traction, and the head was placed in an extended position. After oral and nasal mucosal disinfection with iodine, a latex tube was inserted through the nose for posterosuperior traction of the soft palate and uvula. The anterior atlas arch and the lateral mass joints were exposed, and then the osteophytes and scar tissue between the lateral mass joints and the atlanto-odontoid gaps were removed. After that, satisfactory reduction was achieved under the C-arm X-ray examination. These patients were turned into the prone position with the skull traction and then they were subjected to posterior fixation and fusion.

For patients with posterior-only fixation and fusion, the drainage tube was removed within 2–3 days after the operation, and then they could get up and move around 3–5 days after the operation with the help of collar. The collar was needed to restrict the movement of the craniovertebral junction (CVJ) for at least 2–3 months. For patients with transoral anterior release combined with posterior reduction and fusion, they were needed to be monitored in the intensive care unit for 2–3 days until they were extubated and then transferred to the in-patient ward.

**Fig. 3:**
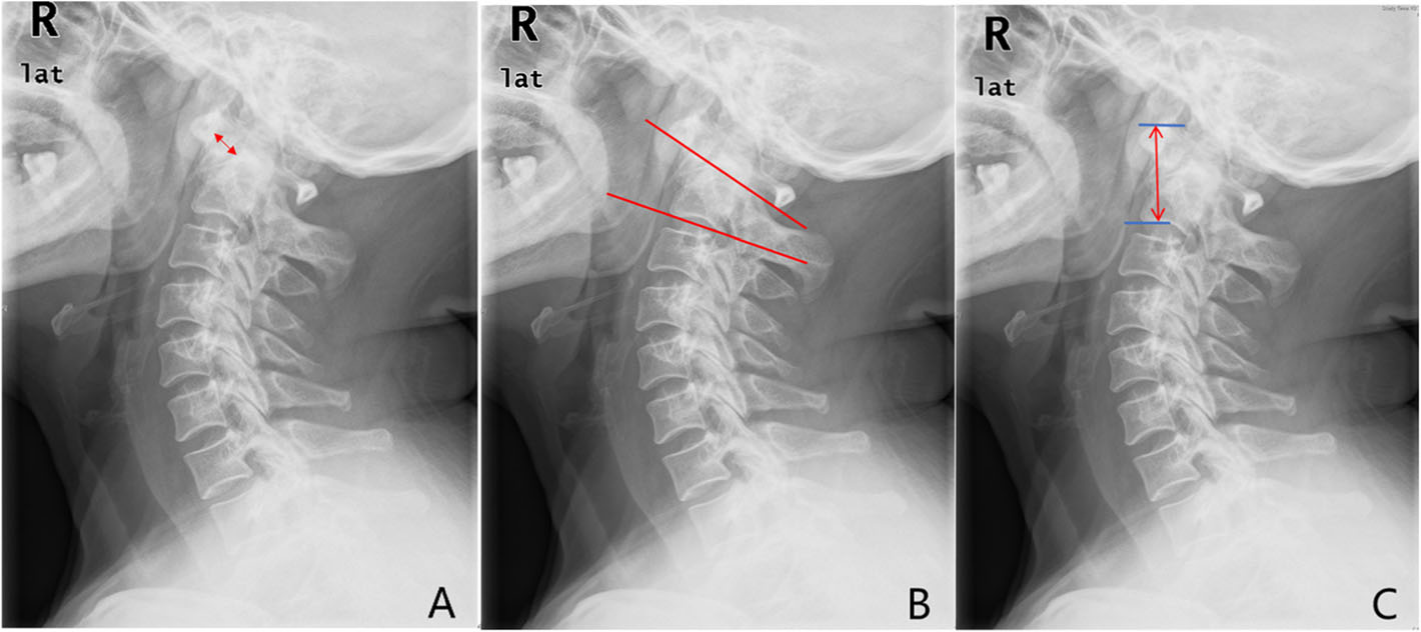
Radiographic parameters. **a** Atlas-dens interval (ADI): the distance between the posterior margins of the anterior arches of the C1 vertebra and the anterior margin of Odontoid process. **b** Atlantoaxial angle (A-A angle, the C1-C2 angle): the angle between the line connecting the lower margins of the anterior and posterior arches of the C1 vertebra and the lower margin of the C2 vertebra. **c** Atlantoaxial height (A-A height): the distance between the upper margin of the anterior arch of the C1 vertebra and the lower margin of the C2 vertebral body

Postoperative cervical X-ray and CT scan were performed to evaluate the fixation and reduction at 1 week after the operation and every follow-up. Follow-up were needed at 3-month, 6-month, 1-year after the surgery and then at yearly intervals. Bony fusion was evaluated at the latest follow-up by cervical CT scan. Radiographic parameters preoperatively, postoperatively and at the latest follow-up, including atlas-dens interval (ADI), atlantoaxial height (A-A height), and atlantoaxial angle (A-A angle, the C1-C2 angle) (Fig. 3) were measured on midline sagittal reconstructed CT images or on a lateral-view plain radiograph [9, 10]. Atlas-dens interval (ADI) was the distance between the posterior margins of the anterior arches of the C1 vertebra and the anterior margin of Odontoid process. Atlantoaxial angle (A-A angle, the C1-C2 angle) was the angle between the line connecting the lower margins of the anterior and posterior arches of the C1 vertebra and the lower margin of the C2 vertebra. Atlantoaxial height (A-A height) was the distance between the upper margin of the anterior arch of the C1 vertebra and the lower margin of the C2 vertebral body. The Japan Orthopedic Association (JOA) scores were needed to assess the clinical outcome improvement.

### Statistical analysis

Clinical data were presented as mean ± SD, and analyzed with IBM SPSS Statistics Version 22.0 (IBM Corp, Armonk, New York, USA). ADI, A-A height, and A-A angle preoperatively and postoperatively, postoperatively and at the latest follow-up were compared using the student’s paired t test. The t-test was used for the comparison and analysis of JOA scores preoperatively and at the latest follow-up. *P* < 0.05 was considered statistically significant.

## Results

There were 86 cases included in this study. With respect to the etiology, there were 9 cases with rheumatoid arthritis, 3 cases with basilar invagination (BI), 5 cases with old odontoid fractures, 11 cases with os odontoideum, 27 cases with acute cervical trauma, and 33 cases with no specific reasons. Preoperative cervical three-dimensional CT revealed occiput (C0)–C1 fusion in 6 case and Os odontoideum in 11 cases. In these cases, high-riding vertebral artery (HRVA) were found in 3 patients, and 1 patient had bilateral HRVA.

Eighty one patients underwent posterior reduction, internal fixation and fusion. 5 patients underwent anterior release, followed by posterior internal fixation and fusion. The mean operative time was 153.9 ± 73.9 min (range 60–385 min), and the mean amount of intraoperative blood loss was 219.1 ± 195.6 mL (range 100-1000 mL). 82 patients got satisfactory postoperative outcomes. Significant neurologic improvement was observed in these patients. Bone fusion was achieved on the midline sagittal reconstructed CT images at the latest follow-up in all these patients except 1 case. All the patients were followed up for 34.84 ± 15.86 months at average (range 12–60 months). The mean ADI value was 7.55 ± 1.67 mm at average preoperatively, and improved to 4.03 ± 1.21 mm postoperatively, and to 4.21 ± 0.99 mm at the latest follow-up. The mean A-A angle was 15.48 ± 9.82 degrees at average preoperatively, and improved to 21.61 ± 10.43 degrees postoperatively, and to 19.73 ± 8.13 degrees at the latest follow-up. The mean A-A height was 35.61 ± 7.66 mm at average preoperatively, and improved to 40.08 ± 8.5 mm postoperatively, and to 38.83 ± 6.97 mm at the latest follow-up. What’s more, the JOA score were 9.6 ± 3.8 preoperatively, and improved to 13.4 ± 3.5 at the latest follow-up, with significant statistical differences. There were significant differences between preoperative and postoperative in the ADI, A-A angle, and A-A height, no significant differences between postoperative and the latest follow-up (Table 2).

**Table 2:** Radiological results

	Pre-operative	Post-operative	Latest follow-up
ADI (mm)	7.55 ± 1.67	4.03 ± 1.21^*1^	4.21 ± 0.99^†1^
A-A angle (degrees)	15.48 ± 9.82	21.61 ± 10.43^*2^	19.73 ± 8.13^†2^
A-A height (mm)	35.61 ± 7.66	40.08 ± 8.50^*3^	38.83 ± 6.97^†3^

*ADI* atlas-dens interval, *A-A angle* atlantoaxial angle, *A-A height* atlantoaxial height

* Paired *t* test between pre-operative and post-operative. Significant differences were considered for *P* value less than 0.05.^*1^P = 0.000, ^*2^P = 0.010, ^*3^P = 0.004

† Paired *t* test between post-operative and final follow-up. Significant differences were considered for *P* value less than 0.05. ^†1^P = 0.079, ^†2^P = 0.292, ^†3^P = 0.224

There were complications in 4 patients. In one patient, C2 pedicle screw misplacement was found on postoperative cervical CT. However, no abnormal symptoms were observed and no revision surgery was done during long-term follow-ups. One patient had breakage of pedicle screw at 4-month after surgery, which needed revision surgery. One patient developed infection after anterior release surgery, and needed repeated wound debridement. One patient died of acute brainstem infarction at two days after surgery.

## Discussion

In our study, we retrospectively investigated clinical outcomes of AAS using different fixation methods with the help of intraoperative skull traction. Skull traction has been widely used for cervical bone fracture, scoliosis correction, the reduction of AAS and et al. Skull traction under general anesthesia, in which the utility of neuromuscular blockade can remove the tension of cervical muscle and ligaments and make the reduction easier. Wang et al. reported the utility of skull traction under general anesthesia in the reduction of AAS. 904 cases were included in their study [2]. Among those 904 cases, 160 cases did not achieve complete reduction on extension radiograph, but were able to be completely reduced following a short-duration of skull traction under general anesthesia [2]. They believed that dynamic radiographs could not reliably reflect reducibility of AAS, but skull traction under general anesthesia could [2]. This method can achieve anatomical reduction through application of substantial traction with total muscle curarization eliminating any muscular resistance under general anesthesia. Dahdaleh et al. previously reported that the utility of neuromuscular blockade and intraoperative traction could overcome the counteractive retractions of the neck muscles and thus facilitated reduction of BI and chronic atlantoaxial rotatory subluxation in pediatric cases [11, 12]. However, skull traction cannot achieve good results in the absence of general anesthesia. Salunke et al. performed conscious cervical traction in their 57 pediatric IAAD patients, but reduction was achieved in only one patient [13]. Kumar carried out conscious cervical traction in 23 children with congenital atlantoaxial dislocation (AAD) with no patient achieving anatomical reduction [14]. In our cases, 81 cases showed satisfactory reduction and 5 cases showed no reduction with the use of intraoperative dynamic imaging and skull traction under general anesthesia. Besides, the maximum of traction weight should be no more than one-sixth to one-fifth of the patient’s body weight. Although skull traction have been believed to be safe under general anesthesia [2, 8], SEPs should be used to monitor neurologic signals.

For AAS without good reduction with the utility of skull traction under general anesthesia, sufficient release of the dislocated atlantoaxial joint is of key importance for successful treatment. In order to achieve good reduction, various methods had been used for IAAD patients, including transoral odontoidectomy and posterior fixation [15], transoral atlantoaxial reduction plate internal fixation [16, 17], transoral atlantoaxial release and posterior internal fixation [18, 19], and anterior submandibular retropharyngeal approach and posterior internal fixation [20]. All these procedures had advantages and disadvantages. Transoral approach can directly release the tight structures around the atlantoaxial joint and even remove the abnormal odontoid compressing the dural sac, which can assist posterior distraction-reduction technique and restore the cranio-cervical anatomy. However, the transoral approach has a high risk of infection [15, 21]. The anterior submandibular retropharyngeal approach is entirely extraoral and extramucosal, which reduces the rate of infection, while it may be difficult for patients with a big body habitus and short neck [20]. In our present study, the anterior release and posterior reduction and internal fixation were performed in 5 AAS patients without good reduction. After anterior atlantoaxial release procedures, good postoperative reduction results were achieved while complications occurred in two of these 5 AAS patients, including one with infection after anterior release surgery and one died of acute brainstem infarction.

The association between the fixation of atlanto-axis and the effect on the alignment of the subaxial cervical spine have attracted people’s attention recently. Yoshimoto H et al. found that when the A-A angle was fixed in an overextended position, the subaxial alignment would correspond to the overextended A-A angle and become kyphotic [22]. Wang et al. have reported that AAD can influence the alignment of the subaxial cervical spine, and achievement of anatomic alignment after the fixation of AAD will allow restoration of the global balance of the cervical spine [2, 23]. When the lower margins of the anterior and posterior arches of the C1 vertebra and the lower margin of the vertebral body of C2 runs almost parallel, the A-A angle is about 30° [24]. When the A-A angle is more than 30°, the C1-C2 region is overextended and the subaxial will become kyphotic. When the A-A angle is less than 30°, the C1-C2 region is distracted and the subaxial alignment will restore lordosis. In our study, the A-A angle in all the patients were fixed less than 30°. No reoperation related to these problems were required in any patient. A long-term study involving a large number of cases is required to further examine the association between the atlantoaxial fixation angle and the change of subaxial alignment.

This study had several limitations. First, this study was a retrospective investigation and was not a randomized case-control study, which may not achieve a strong conclusion. Second, the sample size included in this study was small and this was a single center retrospective study. Third, the etiologies of patients included were multiple. Most of cases included were caused by odontoid fractures, which might attract more attention and limited the results applied to other cases. What’s more, the follow-up time was limited. A longer follow-up, randomized case-control and multi-center study will be needed in the future.

## Conclusion

AAS can cause neck pain and spinal cord compression, even irreversible neurological deficits. Early diagnosis and appropriate treatment should be performed for this kind of abnormity. Intraoperative skull traction can effectively facilitate the surgical procedures for ASS caused by different etiologies. It can not only effectively facilitate the reduction of ASS, but also make the C1 and C2 stable and easy to be implanted during the operation. Satisfactory clinical outcomes can be achieved for patients with satisfactory reduction with the help of intraoperative skull traction under general anesthesia. Multi-center research and longer follow-up are needed in the future to investigate the safety and effectiveness of this method.

## Data Availability

The data used and analyzed during the current study are available in anonymized form from the corresponding author on reasonable request.

## Abbreviations

AAS: Atlantoaxial subluxation
CT: Computed tomography
MRI: Magnetic resonance imaging
BI: Basilar invagination
CTA: Computed tomography angiography
HRVA: High-riding vertebral artery
SEPs: Somatosensory evoked potentials
CVJ: Craniovertebral junction
ADI: Atlas-dens interval
A-A height: Atlantoaxial height
A-A angle: Atlantoaxial angle
JOA: Japan Orthopedic Association
AAD: Atlantoaxial dislocation
